# Two decades of ESKAPE pathogens: Longitudinal analysis of antibiotic resistance trends between 2002 and 2024 in a Hungarian clinical centre

**DOI:** 10.1017/S0950268826101307

**Published:** 2026-03-25

**Authors:** Bence Sajerli, Ágnes Sarkadi-Nagy, Katalin Burián, László Orosz

**Affiliations:** Department of Medical Microbiology, University of Szeged, Hungary

**Keywords:** age-related trends, antimicrobial resistance, clustering, ESKAPE pathogens, instability index

## Abstract

This retrospective study analysed 14,625 isolates of the six major hospital-associated ‘ESKAPE’ pathogens (*Enterococcus faecium, Staphylococcus aureus, Klebsiella pneumoniae, Acinetobacter baumannii, Pseudomonas aeruginosa*, and *Enterobacter* spp.) collected between 2002 and 2024 in a Hungarian tertiary-care centre. Antimicrobial resistance was assessed using the antibiotic resistance index (ARI), multidrug resistance (MDR) ratios, and resistance instability index (RII). *A. baumannii* and *E. faecium* showed the highest resistance burdens and instability. Age showed a significant monotonic association with resistance (Spearman r = 0.88), with peaks in infants, middle-aged women, and the elderly. Species-specific age trends varied, with a negative correlation seen in *Enterobacter* spp. Hierarchical clustering grouped pathogens by resistance trajectory rather than taxonomy. Pairwise resistance distances confirmed divergence between Gram-positive and Gram-negative species. Resistance to aminoglycosides and sulphonamides showed the highest year-to-year variability, as quantified by the RII, particularly in *A. baumannii* and *E. faecium.* Vector autoregressive (VAR) modelling predicted continued MDR increases in these species. A strong correlation was found between ARI and RII (Pearson r = 0.85, p = 0.032). These findings underscore the importance of integrating resistance magnitude and volatility in surveillance.

## Key results and significance

- *Acinetobacter baumannii* and *Enterococcus faecium* exhibited the highest antimicrobial resistance and resistance instability across a 23-year dataset of 14,625 isolates.
- Age was strongly correlated with resistance levels (Spearman r = 0.88), with notable peaks in infants, middle-aged women, and elderly patients.
- Resistance trajectories clustered pathogens independently of taxonomy, suggesting common selective pressures and shared resistance patterns.
- Aminoglycoside and sulphonamide resistance showed the greatest year-to-year variability, particularly in *Acinetobacter baumannii.*
- Vector autoregressive modelling predicted continued multidrug resistance escalation in *A. baumannii* and *E. faecium*, highlighting the need for predictive, species-specific surveillance.

## Introduction

Antimicrobial resistance (AMR) is among the most pressing public health threats globally, as it leads to increased morbidity, mortality, and healthcare costs [1]. Among antibiotic-resistant bacteria, the so-called ‘ESKAPE’ pathogens *– Enterococcus faecium, Staphylococcus aureus, Klebsiella pneumoniae, Acinetobacter baumannii, Pseudomonas aeruginosa*, and *Enterobacter* spp. – represent a group of hospital-associated organisms known for their ability to ‘escape’ the effects of multiple antimicrobial agents [2]. The World Health Organization (WHO) classified these pathogens as priority organisms for new antibiotic development, emphasizing their critical roles in the global AMR crisis [3, 4]. These bacteria employ diverse resistance mechanisms, including efflux pumps, enzymatic degradation of antibiotics, and biofilm formation, allowing them to persist in hospital environments [5]. Infections caused by ESKAPE pathogens are associated with longer hospital stays, increased mortality rates, and limited treatment efficacy, necessitating urgent action in antibiotic stewardship and surveillance [6].

Several studies have reported the increasing drug resistance of ESKAPE pathogens over the past two decades. Laxminarayan et al. demonstrated that resistance among ESKAPE species has intensified across Europe, particularly for carbapenems and fluoroquinolones [7]. Peneş et al. reported a significant increase in multidrug-resistant *P. aeruginosa* over a 5-year period in Romania [8]. Similarly, Ramsamy et al. identified a sharp increase in extended-spectrum β-lactamase (ESBL)-producing *K. pneumoniae* in South African hospitals [9]. Furthermore, Cantón et al. linked the COVID-19 pandemic to a surge in multidrug-resistant infections, likely driven by increased antibiotic use in hospitalized patients [10]. In 2022, our group also analysed resistance trends in Hungary between 2010 and 2020 and detected significant increases in vancomycin-resistant *E. faecium* (VRE) and carbapenem-resistant *A. baumannii* [11].

Beyond resistance magnitude, the temporal stability of resistance patterns may also be epidemiologically relevant. Resistance instability reflects the predictability of resistance dynamics over time; highly variable resistance patterns may indicate fluctuating selection pressures, clonal turnover, or changes in antimicrobial consumption. From a clinical perspective, such instability may reduce the reliability of empirical treatment guidelines and necessitate more frequent antibiogram updates.

Furthermore, analysing cross-species resistance clustering does not imply direct biological interaction between pathogens. Rather, it allows evaluation of whether resistance trajectories converge under shared ecological and therapeutic pressures within the same healthcare environment. If distinct pathogens exhibit parallel resistance dynamics, this may reflect common selective drivers and support coordinated stewardship interventions targeting specific antimicrobial classes.

To address existing gaps in longitudinal AMR research, we conducted a 23-year retrospective analysis of resistance trends in ESKAPE pathogens using clinical microbiology data from a tertiary-care university hospital in Hungary. Despite being a single centre, this institution serves a large, demographically diverse population representative of Central European healthcare systems. Specifically, we characterized long-term pathogen- and drug-specific resistance evolution, examined age-related variability and resistance instability, identified cross-species resistance groupings via clustering, and predicted multidrug resistance (MDR) dynamics using vector autoregressive (VAR) modelling. In addition to describing resistance magnitude, we hypothesized that long-term resistance trajectories may cluster pathogens according to shared ecological and antimicrobial selection pressures within the same healthcare environment rather than strictly by taxonomy. Identifying such convergence may provide insight into common drivers of resistance evolution and support targeted stewardship interventions. By integrating surveillance metrics with predictive modelling and resistance pattern clustering, our study provides transferable insights that could support AMR control strategies in similar healthcare settings across Europe.

## Methods

This study was conducted at the Department of Medical Microbiology, University of Szeged, Hungary. The affiliated clinical microbiology laboratory provides microbiological diagnostic services for the Albert Szent-Györgyi Clinical Center, a 1800-bed primary and tertiary care teaching hospital serving the Southern Great Plain region of Hungary [12]. We applied a retrospective analysis of 14,625 ESKAPE isolates collected between 2002 and 2024 at the University of Szeged, Hungary. No isolate-level deduplication was applied. All clinical isolates recovered during routine diagnostic activity between 2002 and 2024 were included, irrespective of patient identity, sampling frequency, or infection episode. Consequently, multiple isolates from the same patient could be represented in the dataset. Specimen distribution was as follows: urine (25.0%), lower respiratory tract (24.7%), wound/surgical site (20.2%), blood cultures (14.4%), and other sources (15.8%). Sampling reflected routine clinical diagnostic practice. No major structural changes occurred in the hospital’s catchment population during the study period. However, temporary increases in intensive care unit utilization occurred during the COVID-19 period. The dataset included species identification, patient age, and antibiotic susceptibility data, interpreted using Clinical & Laboratory Standards Institute (CLSI) criteria between 2002 and 2011, and from 2012, the European Committee on Antimicrobial Susceptibility Testing (EUCAST) standards. Only antibiotics with ≥10 data points were analysed.

During the study period (2002–2024), antimicrobial susceptibility testing included the agents listed in Table 1.

**Table 1.** Antibiotic panel used for antimicrobial susceptibility testing of ESKAPE pathogens (2002–2024)

Species	Antibiotics tested
*Enterococcus faecium*	Ampicillin; vancomycin; teicoplanin; linezolid; gentamicin
*Staphylococcus aureus*	Cefoxitin (2012–2024); oxacillin (2002–2011); erythromycin; clindamycin; ciprofloxacin; gentamicin; vancomycin; linezolid; trimethoprim–sulfamethoxazole
*Klebsiella pneumoniae*	Ceftriaxone; ceftazidime; cefepime; piperacillin–tazobactam; ciprofloxacin; gentamicin; amikacin; meropenem; imipenem; trimethoprim–sulfamethoxazole
*Acinetobacter baumannii*	Meropenem; imipenem; ciprofloxacin; gentamicin; amikacin; trimethoprim–sulfamethoxazole
*Pseudomonas aeruginosa*	Piperacillin–tazobactam; ceftazidime; cefepime; ciprofloxacin; gentamicin; amikacin; meropenem; imipenem
*Enterobacter* spp.	Ceftriaxone; ceftazidime; cefepime; piperacillin–tazobactam; ciprofloxacin; gentamicin; amikacin; meropenem; imipenem; trimethoprim–sulfamethoxazole

Minor modifications in antibiotic availability occurred in accordance with therapeutic guideline updates; however, only consistently tested antibiotic–species combinations were included in longitudinal analyses.

The annual resistance index (ARI) was calculated as a composite resistance indicator summarizing the proportion of resistant isolates across the tested antibiotic panel for each species in a given year. [13]. For species *s* in year *t*, ARI was defined as:

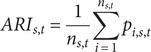

where 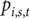 represents the proportion of resistant isolates to antibiotic *i* for species *s* in year *t*, and 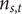 denotes the number of antibiotics tested for that species in that year.

This metric was conceptually adapted from previously described composite resistance indices [13], but differs in that only resistant proportions were included, without weighting of intermediate susceptibility categories. The ARI, therefore, represents an aggregate indicator of resistance burden and does not constitute a standardized international definition.

Species-specific resistance proxy ratios were calculated using clinically dominant marker antibiotics (meropenem for *A. baumannii*, vancomycin for *E. faecium*, ceftriaxone for *Enterobacter* spp. and *K. pneumoniae*, cefoxitin for *S. aureus*, and ciprofloxacin plus meropenem for *P. aeruginosa*). These marker-based ratios were designed to reflect longitudinal trends in clinically relevant resistance phenotypes. This approach does not correspond to standardized international MDR definitions (i.e., non-susceptibility to ≥1 agent in ≥3 antimicrobial categories) and should be interpreted as a resistance proxy rather than a formal MDR classification.

VAR modelling was applied to explore multivariate temporal dynamics in species-specific MDR proxy trajectories. VAR is a multivariate time-series framework in which each variable is expressed as a linear function of its own lagged values and those of other variables, thereby allowing interdependent temporal patterns to be examined [14, 15].

Annual MDR proxy values (2002–2024) for the six ESKAPE pathogens were included as endogenous variables. Model order (lag length) was determined using information criteria (AIC and BIC). Model performance was evaluated using in-sample fit (R^2^) and out-of-sample validation metrics (RMSE and R^2^). Forecasts were generated to explore short-term trajectory tendencies until 2030.

Analyses were performed in Orange Data Mining software (version 3.39.0, Bioinformatics Laboratory, Faculty of Computer and Information Science, University of Ljubljana, Slovenia) and Python (Jupyter Notebook, version 7.2.2), using the statsmodels time-series module for VAR implementation.

VAR was selected over univariate autoregressive models because resistance dynamics across species within a shared healthcare environment may not evolve independently. Forecast uncertainty was not formally quantified, and projections should therefore be interpreted as exploratory rather than predictive.

Hierarchical clustering was applied to longitudinal species-level ARI trajectories (2002–2024) in order to assess similarity in temporal resistance evolution across pathogens. In this context, ‘resistance profile’ refers specifically to the pattern of ARI values over time, rather than a composite of independent phenotypic attributes. The primary objective was to determine whether long-term resistance dynamics group species according to shared ecological and antimicrobial selection pressures rather than taxonomic relatedness. Analyses were performed in Orange Data Mining and Python (Jupyter Notebook, version 7.2.2 [16]), using Ward linkage and Euclidean distance. Dendrograms illustrated species and antibiotic grouping were also generated in Python.

Pairwise species resistance distances were additionally quantified using the Adjusted Rand Index [17], applied here to measure statistical similarity in longitudinal resistance trajectory structure across species. In this context, structural similarity refers to similarity in temporal resistance patterns and clustering behaviour, rather than shared molecular resistance mechanisms. Higher distance values indicate greater divergence in resistance dynamics.

Resistance instability was defined as year-to-year variability in ARI values and quantified using the RII [18]. The RII ranges from 0 (fully stable resistance over time) to 1 (maximally variable resistance) and was calculated as the proportion of annual ARI deviations relative to the total number of observed years:

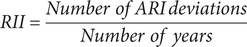



Associations between patient age and species-specific ARI values were assessed using Spearman’s rank correlation coefficient. This non-parametric method was selected because the age distribution was non-normal and the relationship between age and resistance was not assumed to be strictly linear. Spearman’s correlation evaluates monotonic associations without requiring normality assumptions.

Statistical tests (t-test, Mann–Whitney, ANOVA with Tukey post hoc) and visualizations were conducted in GraphPad Prism 10 (GraphPad Software, Boston, MA, USA).

Ethical approval was obtained (BM/27890–3/2024), and the study followed the Declaration of Helsinki.

## Results

### Descriptive epidemiology of ESKAPE isolates

Between 2002 and 2024, 14,625 ESKAPE isolates from 10,323 patients were identified at the Clinical Center of the University of Szeged, Hungary. The most common species were *K. pneumoniae* (26.4%), *P. aeruginosa* (23.1%), and *A. baumannii* (15.1%), followed by *S. aureus* (14.0%), *Enterobacter* spp. (13.3%), and *E. faecium* (8.1%). Urine (25.0%) and lower respiratory tract specimens (24.7%) were the most frequent sources, followed by wound and surgical site (20.2%), blood cultures (14.4%), and miscellaneous sources (15.8%).

### Temporal trends in ESKAPE infections

**Figure 1.**
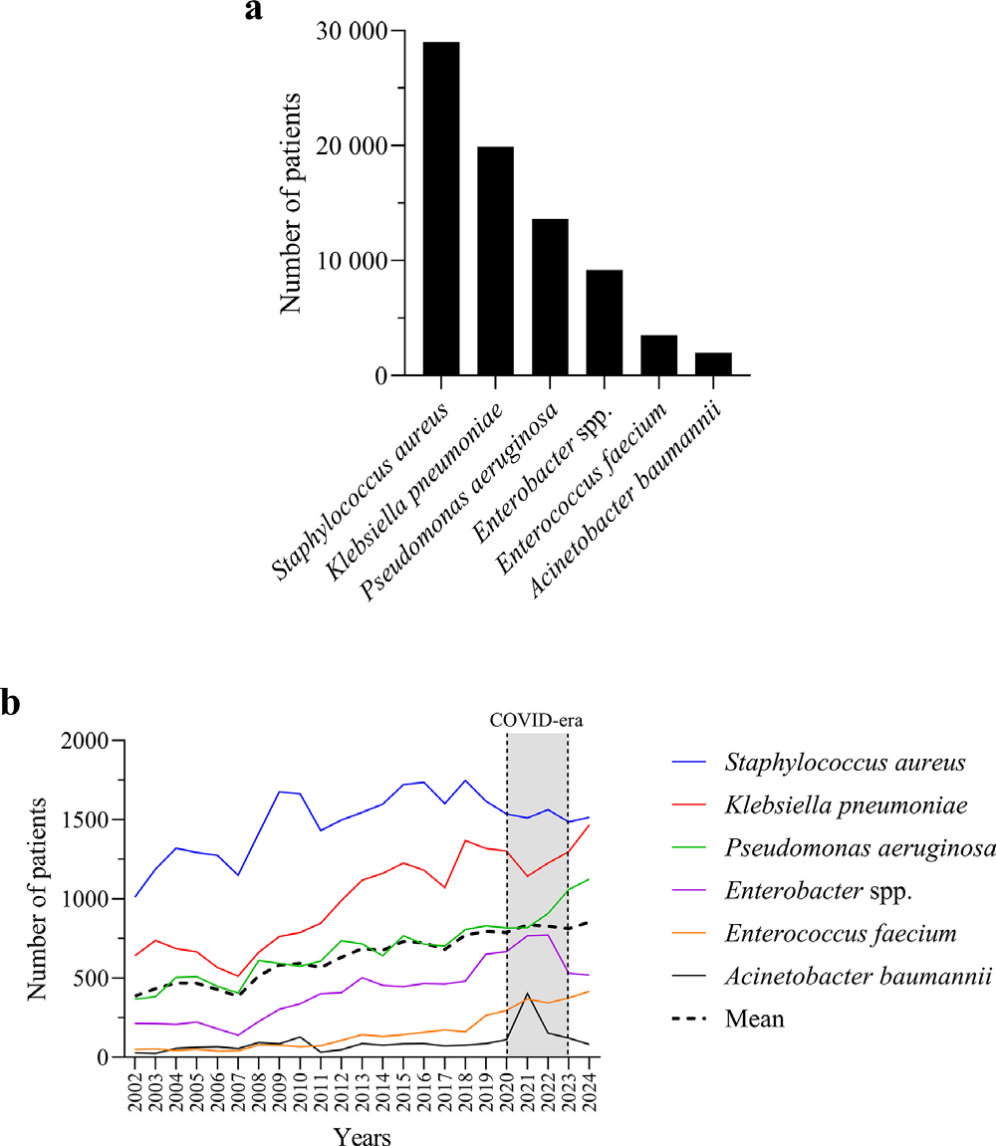
Distribution and temporal trends of ESKAPE pathogens. (a) Total number of patients per pathogen based on all clinical isolates collected between 2002 and 2024. (b) Annual number of patients infected with each ESKAPE species from 2002 to 2024. The dashed line represents the overall mean across species, whereas the shaded area indicates the COVID-19 era.

Between 2002 and 2024, *S. aureus* was the most common ESKAPE pathogen, followed by *K. pneumoniae* and *P. aeruginosa*, while *A. baumannii* remained the least prevalent (Figure 1a). Annual trends (Figure 1b) showed increasing infections with *S. aureus*, *K. pneumoniae*, *P. aeruginosa*, and moderately with *Enterobacter* spp. *E. faecium* remained stable, with a rise from 2018. *A. baumannii* showed a sharp increase in 2021 during the COVID-19 era.

### ARI trends

**Figure 2.**
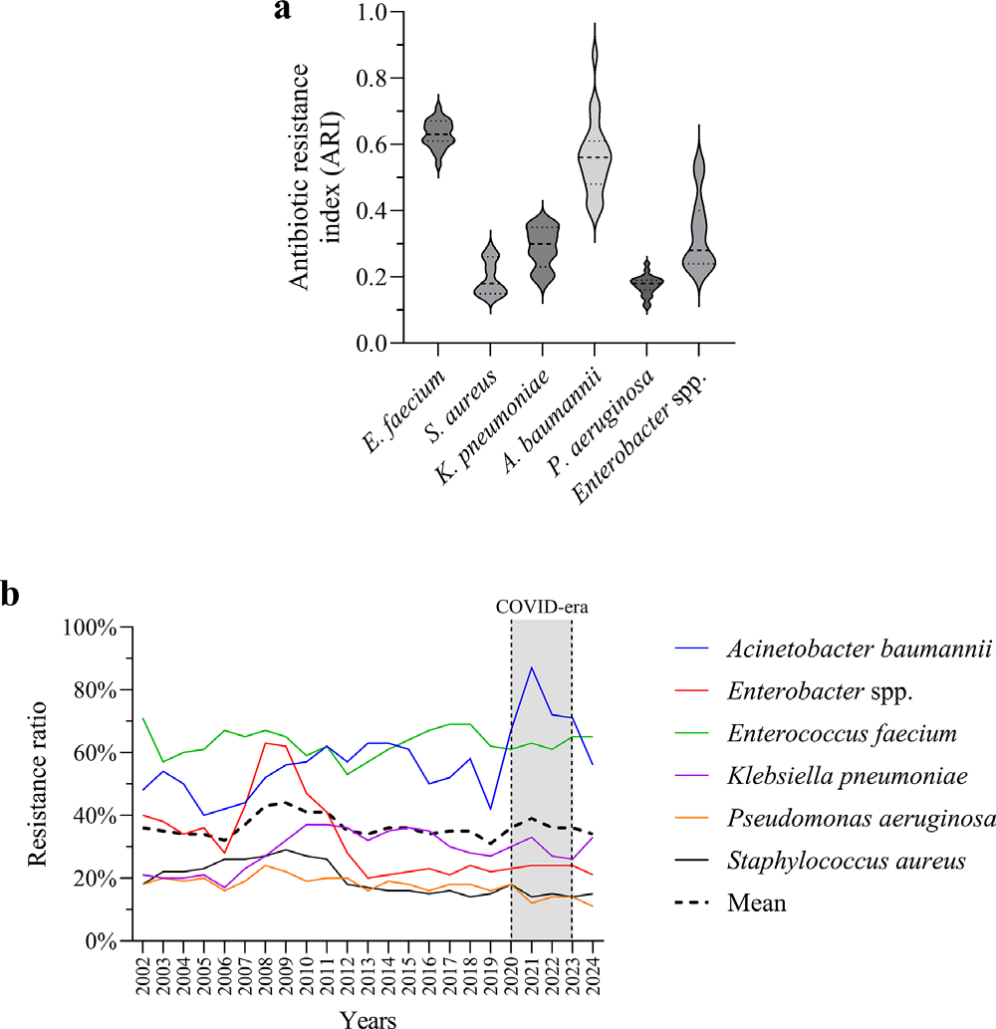
Distribution and temporal trends of antibiotic resistance among ESKAPE pathogens. (a) Violin plots presenting the annual distribution of ARI for each species between 2002 and 2024. The dashed line represents the median, and the shaded area indicates the interquartile range. (b) Annual resistance ratios by species across the study period. The dashed black line represents the average resistance ratio across all ESKAPE species. The shaded area marks the COVID-19 era.

*A. baumannii* showed the highest and most variable ARI values, followed by *E. faecium* and *Enterobacter* spp., confirming their MDR profile (Figure 2a). *K. pneumoniae* had moderate ARI, while *S. aureus* and *P. aeruginosa* showed the lowest resistance levels. Over time (Figure 2b), *A. baumannii* and *E. faecium* maintained high resistance, with *A. baumannii* surging during the COVID-19 era [19]. *K. pneumoniae* resistance gradually increased, whereas *S. aureus* and *P. aeruginosa* remained more stable.

### MDR dynamics

**Figure 3.**
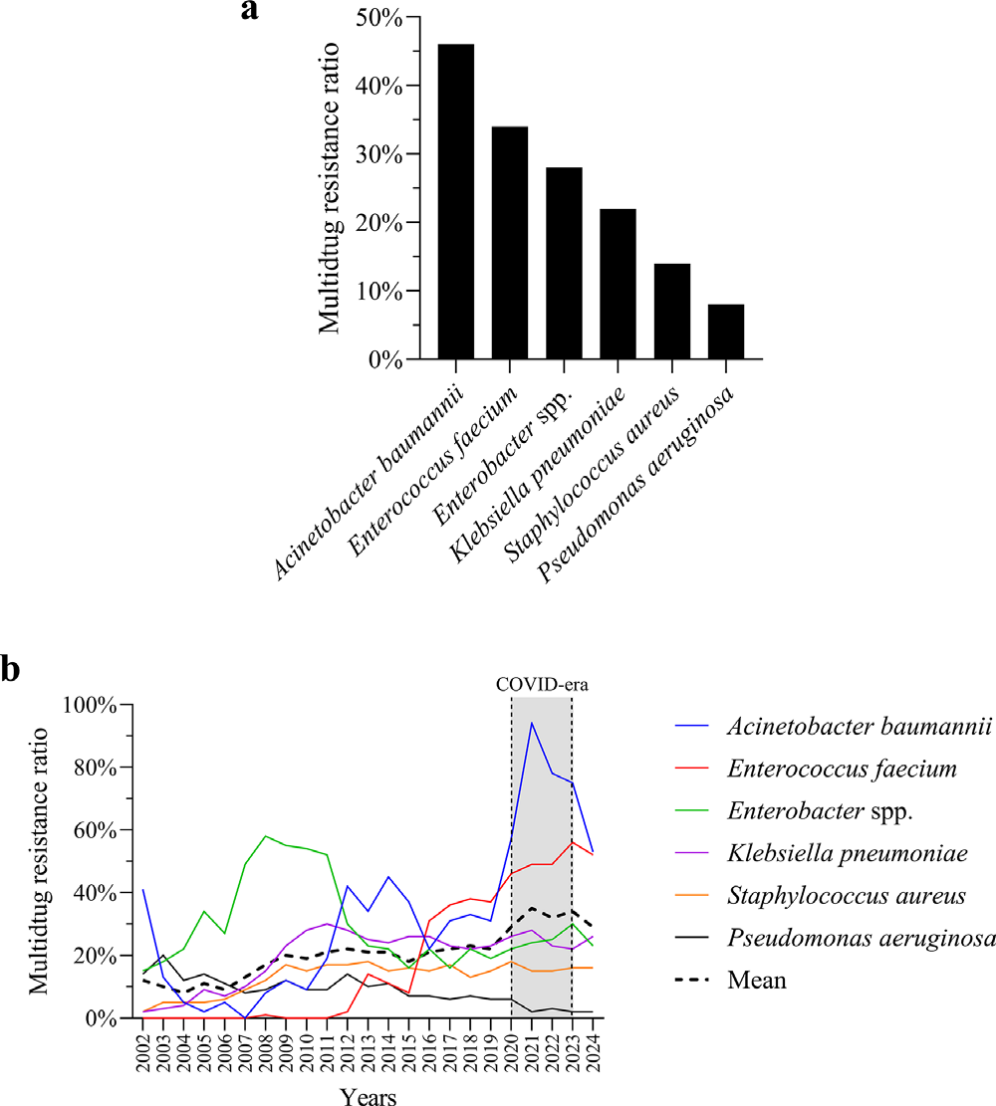
Distribution and temporal dynamics of MDR among ESKAPE pathogens. (a) Bar chart displaying the overall proportion of multidrug-resistant isolates per species. (b) Annual MDR ratios for each species. The dashed black line represents the overall annual mean MDR ratio across all ESKAPE species. The shaded region highlights the COVID-19 era.

MDR prevalence varied markedly among ESKAPE pathogens (Figure 3). *A. baumannii* showed the highest MDR ratio, followed by *E. faecium* and *Enterobacter* spp. (Figure 3a). *K. pneumoniae*, *S. aureus*, and *P. aeruginosa* had lower MDR rates. Longitudinal trends (Figure 3b) showed rising MDR in *A. baumannii* and *E. faecium* from 2019, coinciding with the COVID-19 era. *Enterobacter* spp. fluctuated year to year but showed an overall increase, while *K. pneumoniae*, *S. aureus*, and *P. aeruginosa* remained relatively stable.

### Age-related variability in resistance

**Figure 4.**
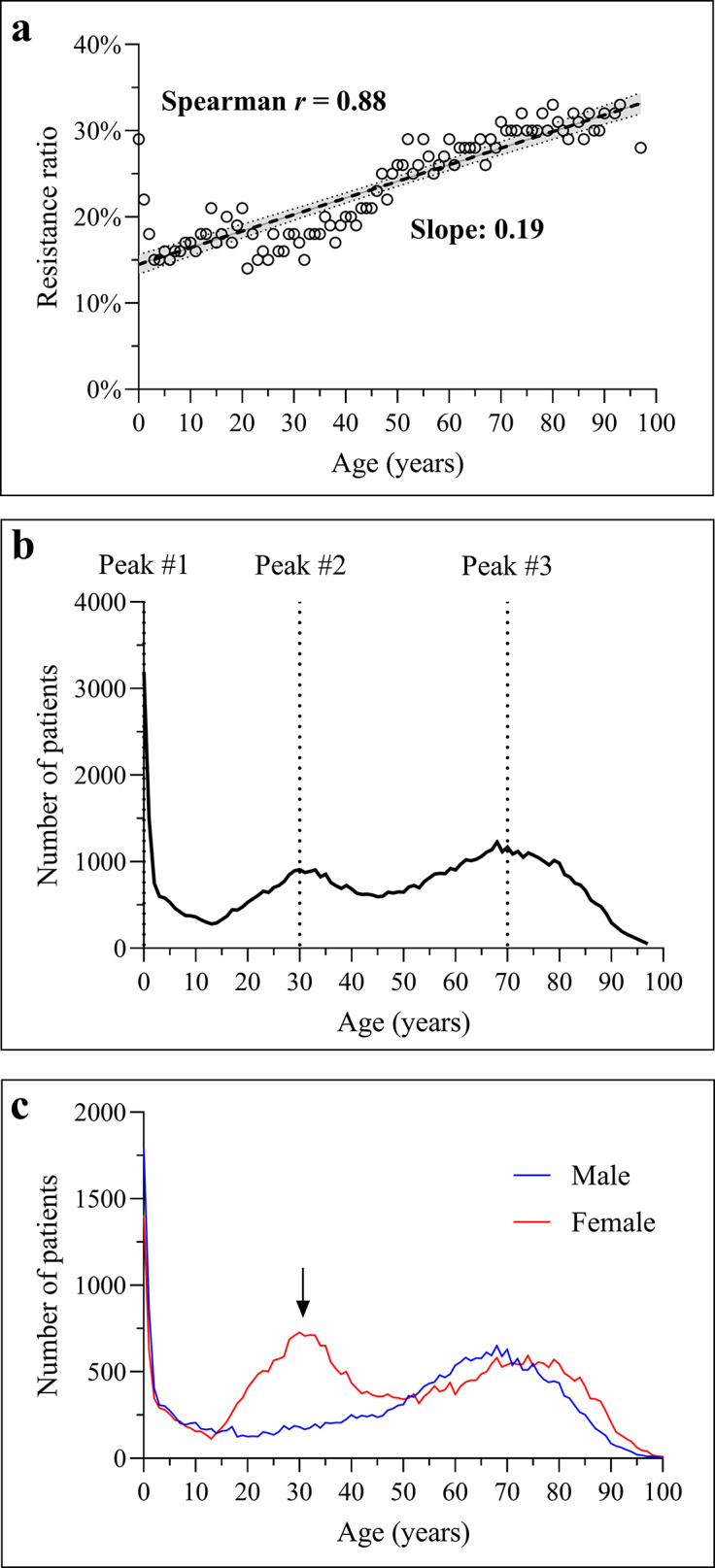
Age distribution of antibiotic resistance and patient counts. (a) Correlation between age and the resistance ratio among all ESKAPE isolates. Each dot represents the mean resistance ratio for a given age (years). A fitted linear regression line with the 95% confidence interval is presented. (b) Age distribution of patients with ESKAPE infections between 2002 and 2024. The plot highlights three major age peaks. (c) Age distribution of infected patients stratified by sex. The arrow highlights the second age peak, around 30 years, at which a distinct distribution pattern is observed.

Resistance increased with age (Spearman r = 0.88; Figure 4a), indicating that older patients are more prone to resistant infections. Age distribution showed a trimodal temporal pattern (Figure 4b), with peaks in infants (0–2 years), middle-aged adults (~30 years), and elderly (~70 years). Notably, in the 30-year group, infections were ~ 3 times more common in women (Figure 4c).

### Species-specific age trends

**Figure 5.**
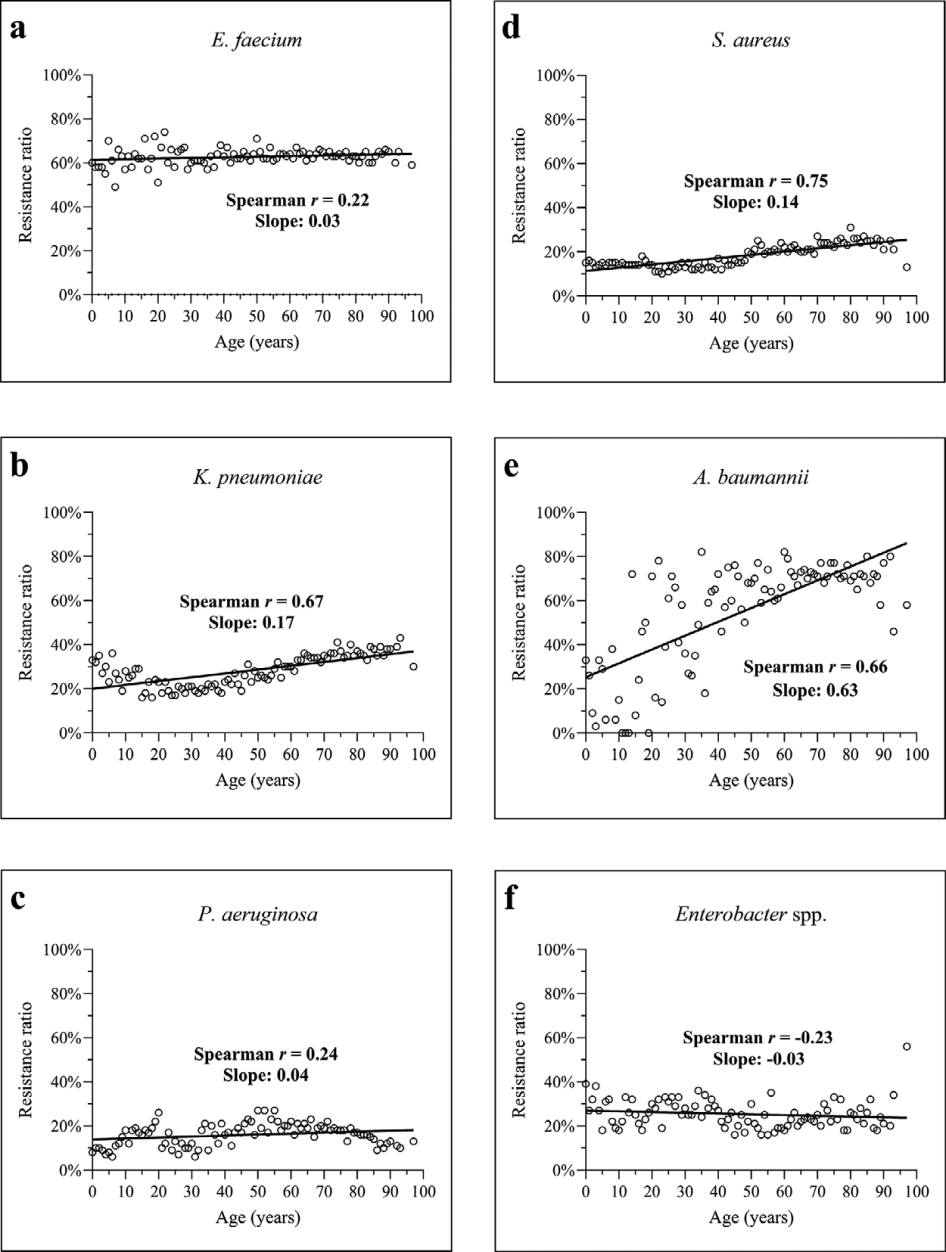
Association between patient age and the antibiotic resistance ratio among ESKAPE pathogens. Spearman’s correlation coefficient (r) and the slope of linear regression models are presented within each panel. Each data point represents the resistance ratio for a given age (years).

Age-related resistance varied across species (Figure 5). *A. baumannii* showed the strongest positive correlation with age (r = 0.66; slope = 0.63), followed by *K. pneumoniae* (r = 0.67; slope = 0.17) and *S. aureus* (r = 0.75; slope = 0.14). *P. aeruginosa* and *E. faecium* had weaker associations (r = 0.24/0.22; slope = 0.04/0.03 respectively). Notably, *Enterobacter* spp. showed a negative correlation (r = −0.23), suggesting higher AMR in younger patients.

### Predicted future trends in MDR

**Figure 6.**
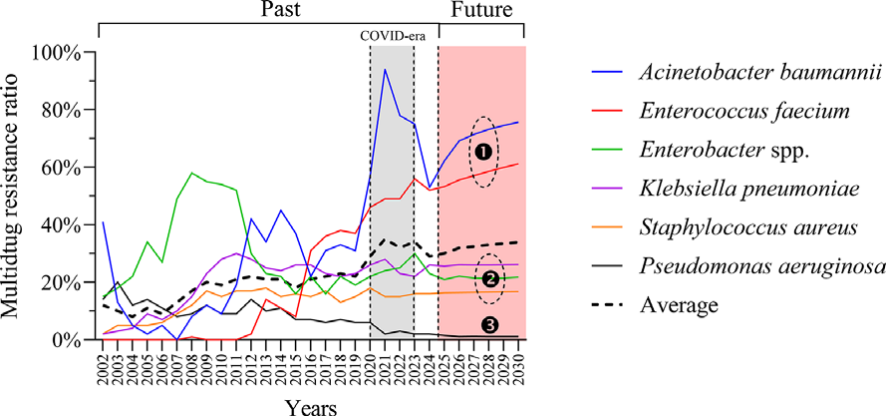
Projected MDR trends in ESKAPE pathogens. Temporal trends in MDR ratios from 2002 to 2024 are presented, with the COVID-19 era highlighted in grey and future projections until 2030 shaded in red. The dashed line represents the mean MDR trend across all species.

VAR modelling was applied to explore potential short-term trajectory patterns of MDR dynamics until 2030 (Figure 6). The model suggested divergent trajectory tendencies across species, with upward trends observed for *A. baumannii* and *E. faecium*, relatively stable patterns for *K. pneumoniae*, *Enterobacter* spp., and *S. aureus*, and lower levels for *P. aeruginosa.* However, model validation showed limited out-of-sample performance (RMSE = 9.837; R^2^ = −1.057) despite good in-sample fit (R^2^ = 0.960), indicating reduced predictive generalizability and potential overfitting (Supplementary Table S1). No formal predictive intervals were estimated. These findings indicate limited predictive performance and are consistent with the stochastic nature of AMR dynamics; projections should therefore be interpreted as exploratory trajectory modelling rather than precise quantitative forecasts.

### Hierarchical clustering of species by ARI

**Figure 7.**
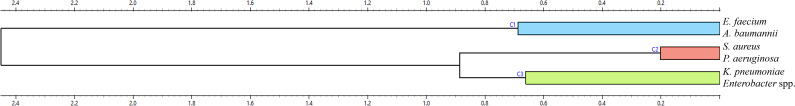
Hierarchical clustering of ESKAPE species based on antibiotic resistance trajectories. The dendrogram displays species-level clustering derived from resistance pattern similarity across antibiotics and years. Branch lengths indicate the relative dissimilarity in resistance profiles.

Hierarchical clustering of species-specific ARI profiles identified three distinct groups (Figure 7). *E. faecium* and *A. baumannii* formed a clearly separated cluster, markedly distant from all other species. This strong divergence reflects their uniquely high resistance burden and temporal instability, consistent with their classification as high-priority MDR pathogens. *S. aureus* and *P. aeruginosa* grouped together in a second cluster, indicating comparable resistance structures despite taxonomic and mechanistic differences. The third cluster comprised *K. pneumoniae* and *Enterobacter* spp., consistent with their shared *Enterobacterales* lineage and overlapping β-lactam resistance mechanisms. While these clusters align with observed ARI dynamics, they partially diverge from predicted MDR trajectories (Figure 6), particularly in the case of *P. aeruginosa*, which, despite its historical clustering with more variable species, is forecasted to remain relatively stable. The clear separation of the *E. faecium-A. baumannii* cluster underscores their distinct evolutionary trajectory and reinforces the need for species-specific intervention strategies.

### Pairwise resistance distance across ESKAPE species

**Figure 8.**
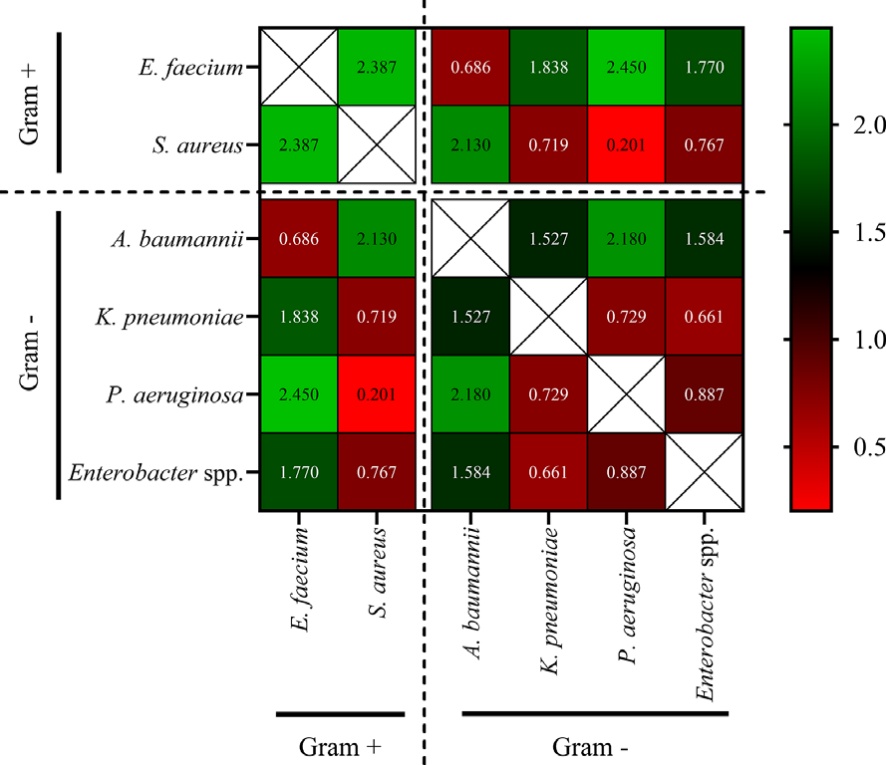
Species-level distance matrix based on antibiotic resistance profiles. The heatmap displays pairwise distance values between ESKAPE species calculated using Adjusted Rand Index-based resistance profile dissimilarities. Higher values (green) indicate greater divergence, whereas lower values (red) reflect higher similarity. The diagonal cells are excluded (white boxes). Species are grouped by Gram status (Gram-positive vs. Gram-negative) as denoted by dashed separators. The colour scale bar reflects resistance distance values from low (red) to high (green).

Adjusted Rand Index-based pairwise distances (Figure 8) confirmed clustering patterns from hierarchical clustering of species-specific ARI (Figure 7). The largest resistance profile differences were between *E. faecium* and *P. aeruginosa* (2.45) and *K. pneumoniae* (1.84). *S. aureus* also diverged strongly from *P. aeruginosa* and *A. baumannii* (both 2.13). In contrast, the smallest distances were between *K. pneumoniae* and *Enterobacter* spp. (0.66), and *A. baumannii* and *K. pneumoniae* (0.73), indicating shared resistance profiles and clinical challenges.

These pairwise distance values broadly corroborate the hierarchical clustering structure observed in Figure 7. The closest resistance similarity was detected between *K. pneumoniae* and *Enterobacter* spp. (distance = 0.66), aligning with their joint clustering in Figure 7. Likewise, *E. faecium* and *A. baumannii* were mutually proximate (0.69), yet strongly divergent from all other species (distances >1.5), further supporting their isolated cluster. Interestingly, *S. aureus* and *P. aeruginosa* showed the highest similarity (0.20), explaining their shared branch in Figure 7 despite their mechanistic and taxonomic differences.

Overall, the ARI-based distance matrix provided quantitative confirmation of the resistance profile groupings derived from hierarchical clustering.

### Hierarchical clustering of antibiotic resistance profiles

**Figure 9.**
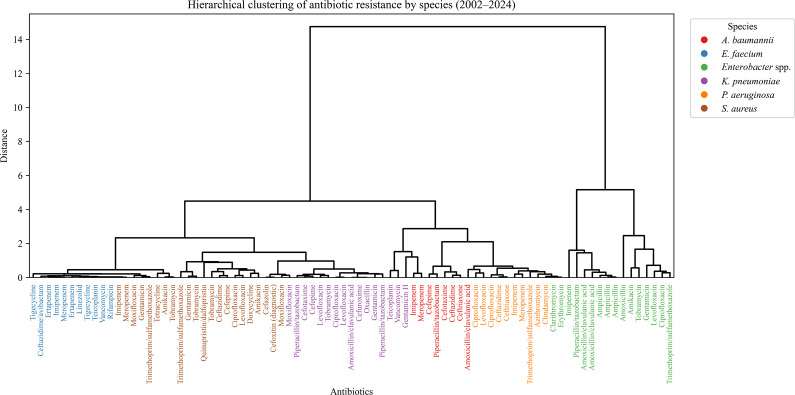
Hierarchical clustering of antibiotic resistance by ESKAPE species. The dendrogram displays the clustering of antibiotics based on resistance similarity across ESKAPE pathogens. Each antibiotic is colour-coded according to its associated species, and branches reflect the degree of dissimilarity in resistance trajectories (distance). Closely clustered antibiotics share similar resistance trends within a species.

Clustering by resistance profiles (Figure 9) revealed species-driven groupings rather than antibiotic class. *E. faecium-*associated agents (e.g. vancomycin, linezolid) formed a compact cluster, reflecting consistent VRE resistance. *K. pneumoniae* antibiotics (e.g. cephalosporins, fluoroquinolones) also clustered tightly, consistent with ESBL-related resistance. Drugs used to treat *Enterobacter* spp. formed a distinct but looser group. In contrast, drugs used against *P. aeruginosa* were widely dispersed, indicating heterogeneous resistance. Antibiotics associated with *A. baumannii* clustered loosely, reflecting diverse mechanisms. *S. aureus-*linked agents showed no clear cluster, highlighting resistance variability.

### Analysis of RII

**Figure 10.**
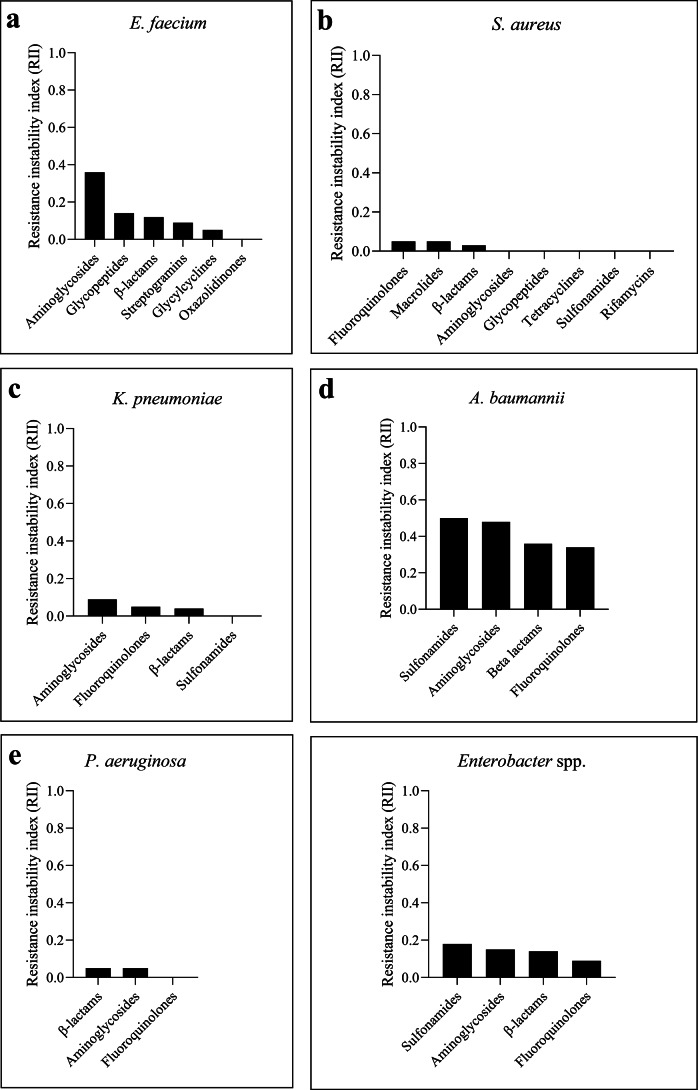
RII by antibiotic class across ESKAPE species. Bar charts depict the RIIs for major antibiotic classes within each ESKAPE species. Higher RIIs indicate greater year-to-year variability in resistance trends. Data are stratified by species.

Temporal RII varied across species and antibiotic classes (Figure 10). *A. baumannii* showed the highest RIIs, especially for sulphonamides, aminoglycosides, β-lactams, and fluoroquinolones, reflecting dynamic resistance shifts. *E. faecium* had moderate-to-high RII, particularly for aminoglycosides and glycopeptides, likely due to clonal turnover and mobile resistance genes. *K. pneumoniae* and *Enterobacter* spp. showed modest RII values, with peaks in aminoglycosides and sulphonamides. In contrast, *S. aureus* and *P. aeruginosa* exhibited low RII across all classes, indicating stable resistance profiles over time.

### Resistance profile divergence by antibiotic class and species

**Figure 11.**
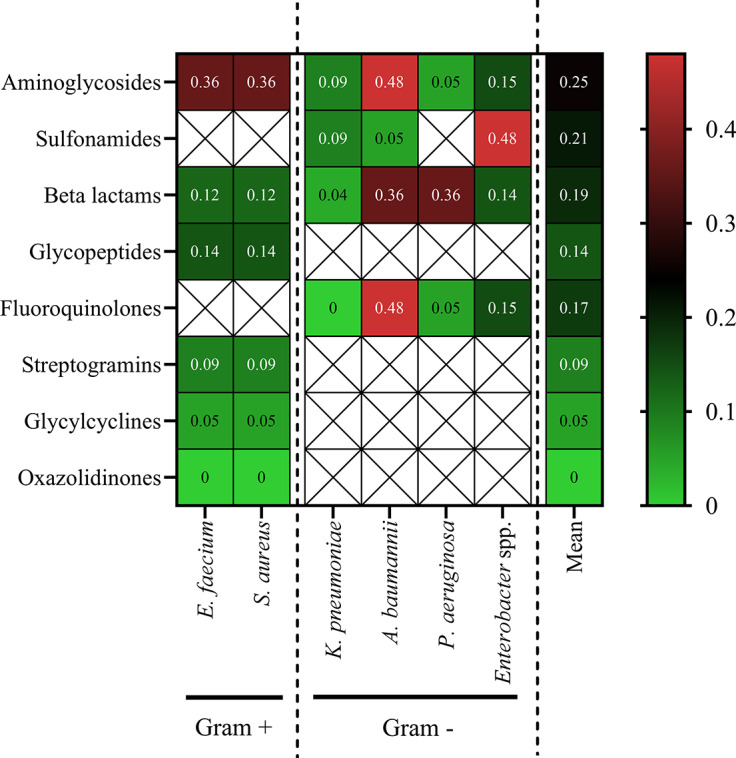
Heatmap of RIIs by species and antibiotic class. The matrix presents RIIs for each ESKAPE species across selected antibiotic classes. Higher RIIs (shaded red) indicate greater year-to-year fluctuations in resistance trends, whereas lower values (shaded green) reflect more stable resistance profiles. Empty cells represent drug-species combinations in which resistance data were not available or not relevant because of intrinsic resistance. The rightmost column summarizes the mean RIIs across all species for each antibiotic class. Gram-positive and Gram-negative species are delineated by dashed lines.

The RII heatmap (Figure 11) revealed the highest instability in aminoglycosides (mean RII = 0.25), particularly in *A. baumannii* (0.48), *E. faecium* (0.36), and *S. aureus* (0.36). Sulphonamides showed moderate volatility, especially in *Enterobacter* spp. (0.48). β-lactam instability was highest in *A. baumannii* and *P. aeruginosa* (both 0.36), and lowest in *K. pneumoniae* (0.04). Fluoroquinolones were generally stable, except in *A. baumannii* (0.48). Other classes (e.g. glycopeptides, oxazolidinones) showed low RIIs, indicating temporal resistance stability.

### Correlation between resistance magnitude and instability

**Figure 12.**
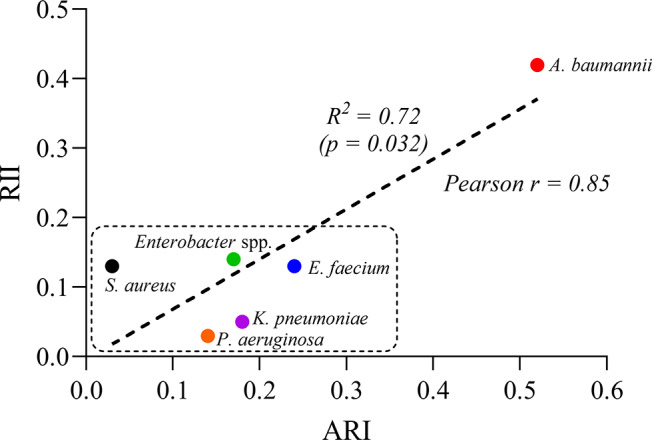
Correlation between ARI and RII across ESKAPE species. Each point represents one species, plotted by its ARI (x-axis) and RII (y-axis). A strong positive correlation was observed (Pearson’s r = 0.85, R^2^ = 0.72, *p* = 0.032), indicating that species with higher resistance burdens tend to exhibit greater temporal resistance variability. *Acinetobacter baumannii* had both high ARI and RII values, whereas most other species clustered in the lower-left region, reflecting lower resistance and instability. The dotted rectangle denotes this low-ARI/low-RII cluster.

A strong positive correlation was found between ARI and RII across ESKAPE species (Pearson’s r = 0.85; R^2^ = 0.72; p = 0.032; Figure 12), indicating that higher resistance levels are associated with greater temporal variability. *A. baumannii* stood out with both high resistance and instability, while other species clustered in the lower-left quadrant, reflecting lower and more stable resistance profiles.

## Discussion

This 23-year longitudinal study provides a comprehensive assessment of AMR among ESKAPE pathogens, elucidating temporal dynamics, age-associated patterns, and resistance clustering. We observed a sustained increase in MDR, particularly among *A. baumannii* and *E. faecium* strains, reinforcing their classification as high-priority pathogens and underscoring the need for species-specific antimicrobial stewardship strategies (Figures 1–3) [20–22].

*A. baumannii* exhibited the highest overall resistance burden (Figure 2). This finding aligns with prior studies that have highlighted the genomic plasticity of this species, propensity for horizontal gene transfer, and ability to form persistent biofilms in hospital environments [20, 23]. The spike in *A. baumannii* prevalence during the COVID-19 pandemic (Figure 2b) likely reflects increased antibiotic use and prolonged intensive care unit stays, consistent with previously reported patterns [24].

These results also resonate with international surveillance data linking *A. baumannii* surges to overwhelmed critical care capacities during pandemics and war-related crises, where broad-spectrum antibiotic use is intensified [25, 26].

Similarly, *E. faecium* showed a steady increase in vancomycin resistance (Figure 2), consistent with global surveillance data and its inclusion on the WHO list of critical MDR pathogens [21, 27]. Both species remain among the leading causes of difficult-to-treat bloodstream infections, ventilator-associated pneumonia, and surgical site infections, especially in intensive care unit settings [22, 28]. Their co-localization in the highest resistance cluster (Figure 7) and co-dominance in resistance variability (Figure 10) further underscore their shared clinical threat profile and justify prioritization in empirical therapy protocols.

Our analysis revealed a statistically significant age-related monotonic trend in resistance (Figure 4), with increasing resistance ratios observed in older patients (Spearman’s r = 0.88). Although the correlation coefficient was high, the absolute differences in resistance proportions across age groups were modest, and therefore, the clinical magnitude of this association should be interpreted cautiously. This trend mirrors previous findings that identified advanced age as a key risk factor for MDR infections, likely due to cumulative healthcare exposure, comorbidities, and immunosenescence [29, 30]. Interestingly, we identified a trimodal age distribution with peaks in infants, middle-aged adults (predominantly women), and older individuals. While the infant peak may reflect empirical antibiotic therapy, the female predominance in middle age warrants further investigation, as it may represent a region-specific demographic pattern. The emergence of a distinct middle-aged female peak may also reflect higher health-seeking behaviour and gynaecological interventions requiring empirical antibiotics, a phenomenon reported in outpatient resistance surveillance studies [31, 32].

Species-specific analysis revealed robust age-associated resistance trends in *A. baumannii*, *K. pneumoniae*, and *S. aureus*, with flatter slopes observed in *E. faecium*, *P. aeruginosa*, and *Enterobacter* spp. (Figure 5) [30, 33]. The negative correlation between age and resistance in *Enterobacter* spp. suggests that younger patients, particularly those in paediatric care, may be disproportionately affected, possibly due to selective pressures in neonatal intensive care units and empirical treatment protocols [34, 35]. This paradoxical finding warrants further genomic investigation to determine whether paediatric-specific clones or mobile resistance elements contribute to this trend.

Forecasting analysis using a VAR model projected continued MDR increases in *A. baumannii* and *E. faecium* through 2030 (Figure 6), consistent with previous findings [11, 36, 37]. Conversely, *K. pneumoniae*, *Enterobacter* spp., and *S. aureus* were predicted to maintain elevated but stable MDR levels, while *P. aeruginosa* was forecast to remain comparatively low. While model performance was satisfactory in-sample (R^2^ = 0.96), out-of-sample accuracy was limited (R^2^ = −1.06), reflecting the inherent complexity and stochasticity of AMR evolution. This limitation highlights the need for hybrid modelling approaches integrating resistance mechanisms, genomic mobility, and healthcare policy shifts. Although VAR modelling enables exploration of interdependent temporal patterns, AMR evolution is influenced by stochastic, policy-driven, and ecological factors that are not fully captured in linear time-series models. No formal predictive confidence intervals were estimated in this analysis. Therefore, projections should be interpreted as exploratory trajectory scenarios rather than deterministic forecasts.

AMR evolution is inherently stochastic and influenced by dynamic factors including antimicrobial consumption, infection control practices, clonal dissemination, and healthcare system pressures. The limited out-of-sample performance of the VAR model and the absence of quantified predictive intervals underscore the uncertainty associated with long-term forecasting. The projected trajectories should therefore be interpreted cautiously and primarily as illustrative scenarios rather than deterministic predictions.

Hierarchical clustering and resistance distance matrices revealed three dominant resistance groupings: (*i*) *A. baumannii* and *E. faecium*; (*ii*) *K. pneumoniae* and *Enterobacter* spp.; and (*iii*) *S. aureus* and *P. aeruginosa* (Figure 7). These clusters reflect taxonomic proximity and shared ecological and antimicrobial selection pressures within the hospital environment. Importantly, the cluster formed by *A. baumannii* and *E. faecium* was completely distinct from the others, highlighting their unique resistance trajectories and clinical importance. Their complete separation in the dendrogram, as evidenced by longer branch lengths and minimal overlap in resistance distance, suggests parallel yet independently driven resistance dynamics shaped by intensive care ecology and high-risk antibiotic exposure. This separation supports their classification as top-priority targets in global AMR mitigation strategies [21, 38, 39]. It is important to note that structural similarity between resistance profiles does not imply shared genetic resistance mechanisms (e.g., efflux pumps or enzymatic degradation), but rather reflects comparable temporal resistance dynamics within the same healthcare environment. From a public health perspective, identifying convergence in resistance trajectories may support coordinated antimicrobial stewardship strategies targeting shared selective pressures, rather than pathogen-specific interventions alone. Although only six pathogens were analysed, formal clustering was employed to provide an objective and reproducible quantification of similarity in longitudinal resistance trajectories, rather than relying solely on descriptive comparison. This approach enhances transparency in comparing relative convergence and divergence across species.

Clustering of antibiotic resistance profiles further demonstrated species-driven patterns (Figure 9). Agents typically used against *E. faecium*, such as linezolid and vancomycin, formed a distinct cluster, as did antibiotics commonly associated with *K. pneumoniae*, including third-generation cephalosporins and fluoroquinolones. *P. aeruginosa*-linked agents exhibited a dispersed pattern, reflecting diverse and often chromosomally encoded resistance mechanisms [40]. The loose aggregation of *P. aeruginosa*-linked antibiotics may reflect its intrinsic resistance via efflux pumps and porin mutations, which produce less predictable co-resistance patterns compared to horizontally acquired resistance determinants.

Analysis of RII showed that *A. baumannii* and *E. faecium* displayed the greatest year-to-year variability, particularly for aminoglycosides, β-lactams, and fluoroquinolones (Figures 10, 11). This instability is likely driven by fluctuating antimicrobial pressure, mobile genetic elements, and dynamic therapeutic protocols [21, 30]. Conversely, *S. aureus* and *P. aeruginosa* exhibited lower RII values, suggesting more stable resistance trajectories largely governed by chromosomal determinants [28]. From a clinical perspective, high RII values could translate to greater unpredictability in empirical therapy outcomes, supporting the implementation of frequent antibiogram updates and rapid diagnostic stewardship in settings with elevated resistance volatility [18].

The strong correlation between ARI and RII (r = 0.85; R^2^ = 0.72) highlights that high resistance prevalence often coexists with elevated temporal variability (Figure 12), particularly in highly adaptable pathogens such as *A. baumannii* [20]. This relationship implies that pathogens with greater evolutionary plasticity are not only more resistant but also more unpredictable in their resistance behaviour over time. This dual challenge further complicates control efforts and necessitates adaptive, dynamic surveillance approaches. From a public health perspective, resistance instability reflects the predictability of resistance dynamics. Pathogens exhibiting high instability may require more frequent surveillance updates and adaptive stewardship strategies, as resistance levels may change more rapidly in response to antimicrobial pressure or clonal turnover. In contrast, stable resistance profiles may allow more consistent empirical treatment strategies. Integrating ARI and RII into routine reporting systems could enable early warning of resistance surges, especially when longitudinal data reveal simultaneous increases in resistance level and fluctuation. These findings emphasize the need for dynamic surveillance frameworks that account for both resistance magnitude and volatility.

This study has several limitations. Its monocentric design may reduce generalizability, although the centre serves a demographically diverse population in Central Europe [12]. The transition from CLSI (2002–2011) to EUCAST (2012–2024) breakpoints, together with subsequent EUCAST revisions, may have influenced susceptibility categorization independently of biological resistance evolution. Although only consistently tested antibiotics were included in longitudinal analyses, temporal changes in antibiotic availability and fluctuations in intensive care case mix may have affected resistance proportions. Because isolate-level deduplication was not performed, repeated sampling from the same patient – particularly in cases of persistent infection, prolonged hospitalization, or outbreak settings – may have led to overrepresentation of specific resistance phenotypes in certain periods. This could have modestly influenced annual resistance proportions. However, given the large sample size and the extended 23-year observation period, the overall longitudinal trends are unlikely to be substantially altered. The use of species-specific marker-based resistance proxies instead of standardized international MDR definitions (non-susceptibility to ≥1 agent in ≥3 antimicrobial categories) may limit comparability with other surveillance studies. For example, cefoxitin-resistant *S. aureus* isolates were captured as part of the resistance proxy but were not automatically assumed to fulfil conventional MDR criteria. These ratios should therefore be interpreted as longitudinal indicators of dominant resistance phenotypes rather than formal MDR classifications. Lastly, while age was strongly associated with resistance, potential confounding variables – such as comorbidities, length of stay, or prior antibiotic exposure – could not be fully assessed. Despite these constraints, the large sample size and extended temporal coverage represent strengths rarely achieved in regional surveillance studies.

In summary, our findings reveal that AMR trajectories among ESKAPE pathogens are species-specific and shaped by ecological, clinical, and evolutionary factors. Quantitative metrics such as ARI and RII provide valuable insights into both resistance burden and instability. These dual indicators may inform predictive dashboards and institutional antibiograms, especially in high-risk wards such as intensive care or transplant units. Incorporating these complementary measures into AMR monitoring frameworks could enhance early warning systems and inform more responsive infection control and stewardship policies. Future interventions should adopt flexible, species-tailored stewardship strategies to address the heterogeneous nature of resistance evolution.

## Supporting information

10.1017/S0950268826101307.sm001 Sajerli et al. supplementary material Sajerli et al. supplementary material

## Data Availability

The anonymized dataset generated and analysed during this study, including annual resistance percentages, ARI, MDR, RII values, and patient demographics, is available from the corresponding author upon reasonable request. Due to institutional policies, raw laboratory records cannot be made publicly available.

## Supplementary material

The supplementary material for this article can be found at http://doi.org/10.1017/S0950268826101307.
