# Supplementary material for: Two decades of ESKAPE pathogens: Longitudinal analysis of antibiotic resistance trends between 2002 and 2024 in a Hungarian clinical centre
Source: Epidemiol Infect. 2026 Mar 25;154:e47. doi: 10.1017/S0950268826101307 (PMC13125272; doi:10.1017/S0950268826101307)
Supplement: Sajerli et al. supplementary material [file S0950268826101307sup001.docx]

**Supplementary Material**

**Supplementary Table S1:** VAR model validation. Model forecasting performance was evaluated using Orange’s Model Evaluation module. A VAR model was trained with 5-fold rolling cross-validation and a 1-step forecast horizon.

| **Metric** | **Value (Out-of-Sample)** | **Value (In-Sample)** |
| --- | --- | --- |
| Root Mean Squared Error (RMSE) | 9.837 | 1.498 |
| Mean Absolute Error (MAE) | 3.425 | 1.027 |
| Mean Absolute Percentage Error (MAPE) | 27.2% | 5.6% |
| Proportion of Correct Direction (POCID) | 42.1% | 76.2% |
| Coefficient of Determination (R²) | –1.057 | 0.960 |
| Akaike Information Criterion (AIC) | N/A | –1.899 |
| Bayesian Information Criterion (BIC) | N/A | 1.275 |
